# Risk factors for epidural anesthesia blockade failure in cesarean section: a retrospective study

**DOI:** 10.1186/s12871-023-02284-w

**Published:** 2023-10-06

**Authors:** Wei-Hsiang Chao, Wen-Shan Cheng, Li-Ming Hu, Chia-Chih Liao

**Affiliations:** 1 Department of Anesthesiology, Chang Gung Memorial Hospital, No.5, Fuxing St., Guishan Dist., Taoyuan City, 33305 Taiwan; 2 College of Medicine, Chang Gung University, Taoyuan City, Taiwan

**Keywords:** Epidural failure, Previous epidural history, Risk factors, Cesarean section

## Abstract

**Background:**

Epidural anesthesia (EA) is the regional anesthesia technique preferred over spinal anesthesia for pregnant women requiring cesarean section and post-operative pain control. EA failure requires additional sedation or conversion to general anesthesia (GA). This may be hazardous during sedation or GA conversion because of potentially difficult airways. Therefore, this retrospective study aimed to determine the risk factors for epidural failure during cesarean section anesthesia.

**Methods:**

We retrospectively analyzed parturients who underwent cesarean section under EA and catheterization at the Chang Gung Memorial Hospital in Taiwan between January 1 and December 31, 2018. Patient data were collected from the medical records. EA failure was defined as the administration of any intravenous anesthetic at any time during a cesarean section, converting it into GA.

**Results:**

A total of 534 parturients who underwent cesarean section were recruited for this study. Of them, 94 (17.6%) experienced EA failure during cesarean section. Compared to the patients with successful EA, those with EA failure were younger (33.0 years vs. 34.7 years), had received EA previously (60.6% vs. 37%), were parous (72.3% vs. 55%), and had a shorter waiting time (14.9 min vs. 16.5 min) (*p* < 0.05). Younger age (OR 0.91, 95% CI 0.86–0.95), history of epidural analgesia (OR 2.61, 95% CI 1.38–4.94), and shorter waiting time (OR 0.91, 95% CI 0.87–0.97) were estimated to be significantly associated with a higher risk of epidural anesthesia failure.

**Conclusion:**

The retrospective study found that parturients of younger age, previous epidural catheterization history, and inadequate waiting time may have a higher risk of EA failure. Previous epidural catheterization increased the risk of EA failure by 2.6-fold compared to patient with no history of catheterization.

## Introduction

Epidural anesthesia (EA) and spinal anesthesia (SA) are regional anesthesia techniques preferred over general anesthesia (GA) in pregnant women who require cesarean section for delivery because of the potentially difficult airway management and systemic effects of GA on the fetus and uterine tone under GA [1, 2]. In comparison to SA, an additional local anesthetic can be administered with EA to prolong the duration of anesthesia. A catheter is also an effective access point for post-operative pain control. Epidural morphine [3, 4] and programmed intermittent epidural bolus [5] would provide adequate analgesia, while post-operative pain control in SA may require additional efforts such as nerve block [6] or intravenous patient-controlled analgesia [7] to prolong the duration of pain control. However, the average failure rate ranges from 13.4 to 22.1% in cesarean sections under EA [8, 9] compared to 0.9–2.5% under SA [10, 11].

Several factors, including patient characteristics and procedural aspects, are associated with an increased risk of epidural failure. High body mass index (BMI), prolonged labor, breakthrough pain during labor analgesia, urgency during cesarean section, an increasing number of top-ups, and maternal height are considered patient-related risk factors [12, 13]. Procedure-related risk factors include anesthesia provided by non-obstetric anesthesiologists, air for loss of resistance, and catheter flexibility [14–16]. Failure of EA requires additional intravenous anesthetics for sedation to achieve an adequate level of anesthesia, or even conversion to GA with endotracheal intubation. This may be hazardous during sedation or GA conversion because of potentially difficult airways.

Therefore, it is necessary to identify the risk factors for epidural failure. This retrospective study aimed to identify risk factors associated with the failure of EA after routine epidural procedures during cesarean delivery.

## Materials and methods

### Patients

This study was approved by the Institutional Review Board of Chang Gung Memorial Hospital, Taoyuan, Taiwan (registration number:201901851B0). All procedures involving human participants were performed in accordance with the ethical standards of the institutional and/or national research committee and the 1964 Helsinki Declaration and its later amendments or comparable ethical standards. The Institutional Review Board of Chang Gung Memorial Hospital waived the need for written informed consent from participants due to the non-interventional study design. We retrospectively analyzed parturient admitted for cesarean section at Chang Gung Memorial Hospital in Taiwan between January 1 and December 31, 2018.

**Fig. 1:**
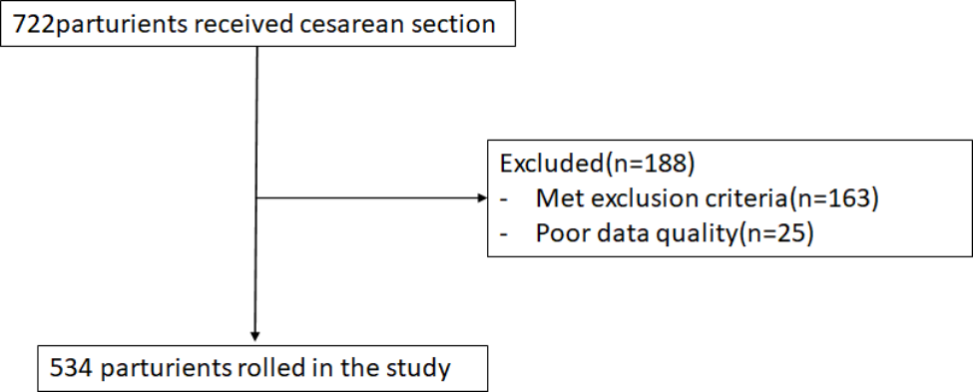
Flow diagram of study participants

Parturients who underwent cesarean section under EA and catheterization in the operating room were included in this study. Parturients were excluded if they had epidural anesthetics other than routine epidural anesthetic mixtures, EA by trainees with experience of lumbar epidural analgesia or anesthesia less than 50 times, short periods between complete anesthesia and surgical incision (less than 10 min), uncertain previous neuraxial anesthesia or analgesia history, conversion from labor epidural analgesia, or history of spine surgery or abnormalities. Patients with known dural punctures before catheterization were also excluded from the study. We retrospectively divided the patients into two groups: epidural failure and non-failure. A total of 722 parturients underwent cesarean section; 163 were excluded as per exclusion criteria, and the quality for 25 was poor. Finally, 534 parturients were enrolled (Fig. 1).

### Study protocol

Routine EA and catheterization were performed using an 18-gauge Tuohy needle and a 20-gauge catheter (Perifix 301 mini set®; B. Braun, Melsungen, Germany). The patient was placed in the right-lateral position during the procedure. After loss of resistance (by air or saline), a 20-gauge catheter was placed in the epidural space, and a 3–5 mL testing dose was injected via catheter. Thereafter, signs of intravascular and intrathecal injections were checked, and if no signs of intravascular or intrathecal injections were observed, the catheter was fixed with adhesive tape. A total 15–24 mL of anesthetic mixture (lidocaine 400 mg, fentanyl 100 mcg, sodium bicarbonate 2.8 g and epinephrine 0.1 mg), including the testing dose, was administered into the epidural space via the catheter. Anesthesia was induced after administration and the time was recorded automatically by a hospital recording system. After preparation for cesarean section, surgeons would start surgery after testing for pinprick sensation. In cases of inadequate anesthesia or block failure, the decision to proceed with EA or switch to GA was made based on the expertise of the anesthesiologist.

### Data collection and variable definition

Patient data were collected from the medical records. Demographic characteristics including age, height, weight, BMI, history of previous epidural anesthesia or analgesia, puncture site, loss of resistance skill, procedure performance (visiting staff or experienced trainees), volume of anesthetic mixture administered, emergency or scheduled cesarean section, rupture of membranes before surgery, and American Society of Anesthesiologists physical status were recorded. EA failure was defined as the administration of any intravenous anesthetic at any time during a cesarean section, converting it into sedation or GA. Intravenous anesthetics included thiamylal, propofol, ketamine, midazolam, and fentanyl. Waiting time, defined as the duration between EA completion and surgical incision was also collected from medical records. The primary outcome of our study was the failure of EA and the secondary outcomes were the identified risk factors for EA failure.

### Statistical analysis

The baseline characteristics of the participants in the two groups (failure of EA vs. non-failure, and previous epidural analgesia vs. none) were compared using an independent sample t-test for continuous variables or a chi-square test for categorical variables. The association between clinical characteristics and risk of anesthesia failure was investigated using univariate and multivariable logistic regression analyses. Factors potentially correlated with the risk of anesthesia failure were initially screened using a series of univariate logistic regression models. Factors with a significance level of less than 0.2 in the univariate analyses were further analyzed using a multivariable model [17]. The survival rate from the time to intravenous anesthetic supplementation was estimated using the Kaplan-Meier method, along with the log-rank test to compare the groups (previous epidural analgesia vs. none). To evaluate potential bias, the dominant risk factors (previous epidural analgesia vs. none) were compared using an independent sample t-test for continuous variables or a chi-square test for categorical variables. All tests were 2-tailed and *p* < 0.05 was considered statistically significant. Data were analyzed using SPSS version 25 (IBM SPSS Inc, Chicago, Illinois, USA).

## Results

### Baseline characteristics

In total, 722 parturients were initially considered for this study. After excluding 188 patients, 534 parturients were included (Fig. 1). Of these, 94 (17.6%) experienced EA failure during cesarean section (Table 1). The mean age was calculated to be 34.4 years. A total of 220 parturients (41.2%) had previously received epidural EA, and the most frequent puncture site was L3-4 (n = 442, 82.8%). Most loss-of-resistance methods used air (n = 457, 85.6%). A total of 310 (58.1%) patients were parous. The mean waiting time was 16.2 min. Compared to the patients with successful EA, those with EA failure were younger (33.0 years vs. 34.7 years) and more likely to have received EA previously (60.6% vs. 37%), were parous (72.3% vs. 55%), and had a shorter waiting time (14.9 min vs. 16.5 min) (*p* < 0.05).

**Table 1:** Baseline characteristics of study subjects according to epidural failure

Variable	Total (*n* = 534)	Epidural failure		
		No (Success) (*n* = 440)	Yes (Failure) (*n* = 94)	*P* value
Age, years	34.4 ± 4.9	34.7 ± 4.8	33.0 ± 5.3	0.003^a^
Height, cm	159.1 ± 5.8	159.1 ± 6.0	159.0 ± 4.7	0.841^a^
Weight, kg	71.2 ± 12.1	71.6 ± 12.1	69.4 ± 11.9	0.106^a^
Body mass index, kg/m^2^	28.1 ± 4.6	28.3 ± 4.6	27.4 ± 4.3	0.091^a^
Previous epidural analgesia	220 (41.2)	163 (37.0)	57 (60.6)	< 0.001^b^
Puncture site				0.314^b^
L2-3	69 (12.9)	54 (12.3)	15 (16.0)	
L3-4	442 (82.8)	369 (83.9)	73 (77.7)	
L4-5	23 (4.3)	17 (3.9)	6 (6.4)	
Loss of resistance methods				0.885^b^
Air	457 (85.6)	377 (85.7)	80 (85.1)	
Saline	77 (14.4)	63 (14.3)	14 (14.9)	
Catheter depth, cm	5.3 ± 0.7	5.24 ± 0.65	5.31 ± 0.67	0.393^a^
Experience of anesthesia provider				0.725^b^
Resident	444 (83.1)	367 (83.4)	77 (81.9)	
Obstetric anesthesiologist	90 (16.9)	73 (16.6)	17 (18.1)	
Emergency surgery	217 (40.6)	185 (42.0)	32 (34.0)	0.152^b^
Rupture of membrane	76 (14.2)	65 (14.8)	11 (11.7)	0.439^b^
Parity				0.002^b^
Nulliparous	224 (41.9)	198 (45.0)	26 (27.7)	
Parous	310 (58.1)	242 (55.0)	68 (72.3)	
Waiting time	16.2 ± 4.7	16.5 ± 4.7	14.9 ± 4.7	0.002^a^

Data were presented as frequency (percentage) or mean ± standard deviation

“a” indicates independent sample t-test and “b” represents chi-square test

### Associated factors for failure of analgesia

**Fig. 2:**
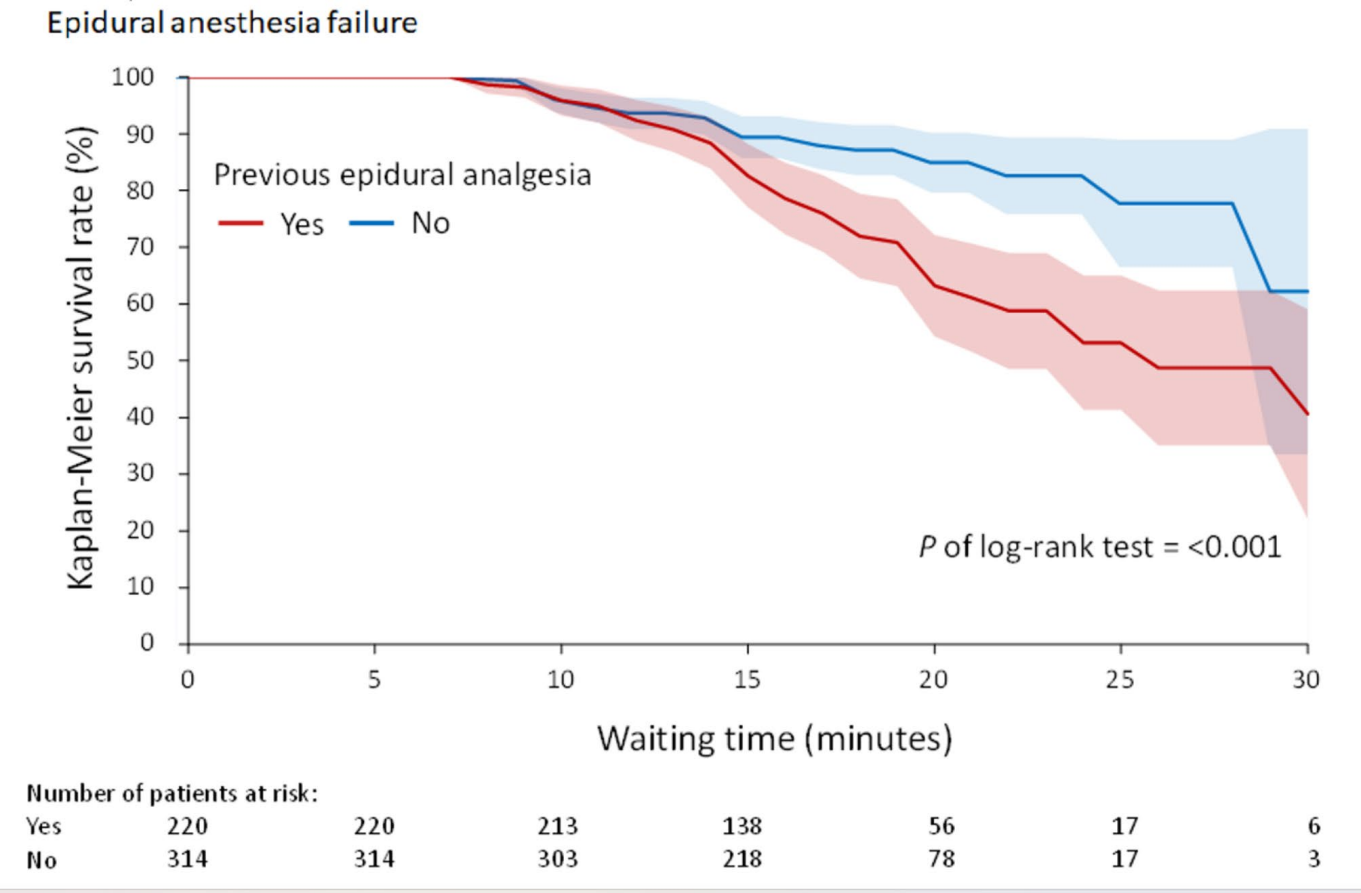
**The Kaplan-Meier survival rate of time to epidural anesthesia failure in the patients with and without previous epidural analgesia** The survival rate of paturients with previous epidural analgesia drops quicker than those without previous analgesia experience after 12 min of epidural anesthetic administration

Univariate logistic regression models showed that the following factors were potentially correlated with the risk of epidural anesthesia failure: younger age, lower BMI, history of epidural analgesia, emergency surgery, parity, and shorter waiting time. A multivariable model was used to rule out correlations that may affect risk factors. The multivariable model demonstrated that younger age (odds ratio [OR] 0.91, 95% confidence interval [CI] 0.86–0.95), history of epidural analgesia (OR 2.61, 95% CI 1.38–4.94), and shorter waiting time (OR 0.91, 95% CI 0.87–0.97) were significantly associated with a higher likelihood of EA failure (Table 2). The time to epidural failure among participants who previously received EA and those who did not are shown in Fig. 2. Patients with history of epidural analgesia had a greater risk of epidural failure within a shorter period.

**Table 2:** The associated factors of epidural failure

Variable	Univariate analysis			Multivariable analysis	
	Crude OR (95% CI)	*P* value		Adjusted OR (95% CI)	*P* value
Age, years	0.94 (0.89–0.98)	0.004		0.91 (0.86–0.95)	< 0.001
Body mass index, kg/m^2^	0.96 (0.91–1.01)	0.092		0.96 (0.90–1.01)	0.112
Previous epidural analgesia	2.62 (1.66–4.13)	< 0.001		2.61 (1.38–4.94)	0.003
Puncture site					
L2-3	1.00				
L3-4	0.71 (0.38–1.33)	0.287			
L4-5	1.27 (0.43–3.79)	0.667			
Loss of resistance methods (Saline vs. Air)	1.05 (0.56–1.96)	0.885			
Catheter depth, cm	1.16 (0.82–1.64)	0.392			
Experience of anesthesia provider (Obstetric anesthesiologist vs. Resident)	1.11 (0.62–1.99)	0.725			
Emergency surgery	0.71 (0.45–1.14)	0.153		0.83 (0.50–1.35)	0.446
Rupture of membrane	0.76 (0.39–1.51)	0.440			
Parity (Parous vs. Nulliparous)	2.14 (1.31–3.49)	0.002		1.44 (0.73–2.84)	0.289
Waiting time	0.92 (0.87–0.97)	0.002		0.91 (0.87–0.97)	0.002

Abbreviations: OR, odds ratio; CI, confidence interval

### Characteristics of patients with and without previous epidural analgesia

Based on the aforementioned analyses, previous epidural analgesia appears to be the dominant factor responsible for epidural failure. Therefore, we compared the clinical characteristics of patients with and without previous epidural analgesia. The results showed that the patients with previous epidural analgesia were older (35.4 years vs. 33.7 years), less likely to have emergent surgery (31.4% vs. 47.1%), had a lower incidence of rupture of membrane (9.1% vs. 17.8%), and were more likely to be parous (95.9% vs. 31.5%), compared to those without previous epidural analgesia (*p* < 0.05) (Table 3).

**Table 3:** Baseline characteristics of study subjects according to previous epidural analgesia

Variable	Previous epidural analgesia		
	Yes (*n* = 220)	No (*n* = 314)	*P* value
Age, years	35.4 ± 4.4	33.7 ± 5.2	< 0.001
Height, cm	159.4 ± 5.2	158.9 ± 6.2	0.425
Weight, kg	71.2 ± 11.6	71.3 ± 12.4	0.907
Body mass index, kg/m^2^	28.0 ± 4.0	28.2 ± 4.9	0.508
Puncture site			0.089
L2-3	33 (15.0)	36 (11.5)	
L3-4	182 (82.7)	260 (82.8)	
L4-5	5 (2.3)	18 (5.7)	
Loss of resistance methods			0.412
Air	185 (84.1)	272 (86.6)	
Saline	35 (15.9)	42 (13.4)	
Catheter depth, cm	5.23 ± 0.65	5.28 ± 0.66	0.357
Experience of anesthesia provider			0.357
Resident	179 (81.4)	265 (84.4)	
Obstetric anesthesiologist	41 (18.6)	49 (15.6)	
Emergency surgery	69 (31.4)	148 (47.1)	< 0.001
Rupture of membrane	20 (9.1)	56 (17.8)	0.004
Parity			< 0.001
Nulliparous	9 (4.1)	215 (68.5)	
Parous	211 (95.9)	99 (31.5)	
Waiting time	16.3 ± 4.9	16.2 ± 4.6	0.874
epidural failure	57 (25.9)	37 (11.8)	< 0.001

Data were presented as frequency (percentage) or mean ± standard deviation

## Discussion

In this study, we examined the risk factors that may cause failure in the conversion of labor analgesia to cesarean delivery anesthesia reported in previous studies. These include procedures performed by trainees, parturients with high BMI, and using air for the loss of resistance test [18, 19]. However, no significant differences were observed between the groups in the present study. For epidural catheterization performed during the study period, all trainees who performed EA had experience with epidural procedures, including cesarean section and labor analgesia in over 50 cases. This retrospective study showed a higher failure rate of EA in parturients with history of epidural analgesia, younger age, and insufficient waiting time before surgical incision. This may result in conversion to GA and the management of difficult airways.

Previous studies have indicated that a higher BMI leads to both technical difficulty and failure of neuraxial anesthesia [20], whereas a higher risk of extension failure to surgical anesthesia has been reported in obese parturients [21]. However, in this study, the number of parturients with a high BMI was small; only 5 parturients out of 539 had a BMI ≥ 40 kg/m^2^, which is defined as morbid obesity. In contrast, owing to the physical limitations of the epidural needle, which is only 8 cm in length, a patient with high BMI may experience epidural catheterization failure and may be required to switch to spinal anesthesia, causing difficulties in tracing anesthesia records. Therefore, fewer parturients with high BMI were included in the present study.

Loss of resistance to air may increase the risk of epidural failure compared to saline [22, 23]. It was mentioned that the air might affect the spread of local anesthetic, resulting in an incomplete “patchy block”, leading to increased use of intraoperative intravenous anesthetics. Segal examined 929 labor anesthetics and reported no difference between air and saline when the preferred technique was used [24]. In this study, the failure rate with air was 18%, whereas that with saline was 17%, with no significant difference. The loss of resistance skill with air was mainly used at Chang Gung Memorial Hospital, and repeated tests with air were avoided in the protocol, which may have resulted in a slightly higher failure rate in the air group than that in the saline group; however, no significant difference was observed in the statistical analysis.

Parturients with previous epidural experience had a higher failure rate than those receiving EA for the first time. Previous studies have reported significant inflammatory changes and adhesions in patients with a history of EA using an epidural scope [25, 26]. Puncture of the flava ligament and epidural catheterization lead to congestion and adhesions in the epidural space, resulting in disturbance in the spread of local anesthetic in the epidural space [27]. Traumatic changes such as fibrosis, congestion and hemorrhage may influence the local cephalic spread of anesthetics, and worsen drug penetration leading to top-up failure or inadequate blockade. Repeated epidural anesthesia is associated with a higher risk of unilateral block [28].

As shown in Fig. 2, the survival curve after administration of epidural anesthetics was almost the same before 12 min, suggesting that the failure resulted mainly from inadequate waiting time for lidocaine-bicarbonate-epinephrine-fentanyl to reach surgical anesthesia at the T7 level [29, 30]. The survival rate dropped much quicker after 12 min epidural anesthetic administration in parturients with history of epidural analgesia than in those with no such history, indicating a difference between parturients with and without a history of epidural analgesia. Patients with a history of epidural analgesia showed a higher failure rate during the same waiting period. As found in previous studies [24, 25], the cephalic spread of local anesthetic is slower in parturients with previous epidural experience, which also affects drug penetration to nerve roots, leading to inadequate anesthesia during surgery. Such parturients need extra intravenous anesthetic administration to fulfil anesthesia needs and even require switching to GA.

Age also plays a role in epidural failure. Younger patients may have a higher risk of epidural failure. Several studies have also reported younger age as a risk factor of epidural failure [14, 31]. We suggest that, because of the decrease in myelinated fibers associated with aging [32], it takes more time for local anesthetics to penetrate the nerve roots and achieve adequate anesthesia for cesarean section. However, further studies are required to evaluate these risk factors.

A limitation of this study was its retrospective design. Failure was defined as an inadequate block and the inability to perform a cesarean section without intravenous anesthetic administration; however, the records did not indicate whether it was an inadequate block or if the parturient was administered a sedative due to nervousness. To avoid bias, the procedures should be performed by obstetric anesthesiologists and loss of resistance test must be performed only using saline or air. Further prospective studies with additional control factors are warranted to clarify these aspects.

## Conclusions

This retrospective study revealed that patients with history of epidural catheterization, inadequate waiting time, and younger age may have a higher risk of EA failure. Previous epidural catheterization increased the risk of EA failure by 2.6-fold compared to patients with no history of catheterization. Therefore, we suggest that parturients with previous epidural history and suspected difficult airways may consider spinal or combined spinal-epidural anesthesia for cesarean section.

## Data Availability

All data generated or analyzed during this study are included in this published article.

## Abbreviations

EA: Epidural anesthesia
GA: General anesthesia
OR: Odds ratio
CI: Confidence interval
SA: Spinal anesthesia
BMI: Body mass index
